# Longitudinal evaluation of polyneuropathy in Parkinson’s disease

**DOI:** 10.1007/s00415-024-12579-8

**Published:** 2024-07-26

**Authors:** Eun Hae Kwon, Antonia Bieber, Paula Schülken, Katharina Müller, Eva Kühn, Paulina Averdunk, Saskia Kools, Lovis Hilker, András Kirchgässler, Lea Ebner, Louisa Ortmann, Louisa Basner, Julia Steininger, Teresa Kleinz, Jeremias Motte, Anna Lena Fisse, Christiane Schneider-Gold, Ralf Gold, Raphael Scherbaum, Siegfried Muhlack, Lars Tönges, Kalliopi Pitarokoili

**Affiliations:** 1 Department of Neurology, St. Josef-Hospital, Ruhr University Bochum, Bochum, Germany; 2 Neurodegeneration Research, Centre for Protein Diagnostics (ProDi), Ruhr University, 44791 Bochum, Germany

**Keywords:** Parkinson’s disease, Polyneuropathy, Large fiber neuropathy, Nerve conduction study, Progression

## Abstract

**Background:**

Increasing evidence indicates a higher prevalence of polyneuropathy (PNP) in Parkinson’s disease (PD). However, the involvement of large fiber neuropathy in PD still remains poorly understood. Given the lack of longitudinal data, we investigated the course of PNP associated with PD.

**Methods:**

In total, 41 PD patients underwent comprehensive clinical evaluation including motor and non-motor assessments as well as nerve conduction studies at baseline and at 2 years of follow-up. The definition of PNP was based on electrophysiological standard criteria. Common causes of PNP were excluded.

**Results:**

At baseline, PNP was diagnosed in 65.85% of PD patients via electroneurography. Patients with PNP presented with higher age (*p* = 0.019) and PD motor symptom severity (UPDRS III; *p* < 0.001). Over the course of 2 years, PNP deteriorated in 21.95% of cases, and 26.83% remained without PNP. Deterioration of nerve amplitude was most prevalent in the median sensory nerve affecting 57.58% of all PD cases with an overall reduction of median sensory nerve amplitude of 45.0%. With regard to PD phenotype, PNP progression was observed in 33.33% of the tremor dominant and 23.81% of the postural instability/gait difficulties subtype. Decrease of sural nerve amplitude correlated with lower quality of life (PDQ-39, *p* = 0.037) and worse cognitive status at baseline (MoCA, *p* = 0.042).

**Conclusion:**

The study confirms the high PNP rate in PD, and demonstrates a significant electrophysiological progression also involving nerves of the upper extremities. Longitudinal studies with larger cohorts are urgently needed and should elucidate the link between PD and PNP with the underlying pathomechanisms.

**Supplementary Information:**

The online version contains supplementary material available at 10.1007/s00415-024-12579-8.

## Introduction

As the most common neurodegenerative movement disorder [1], the high clinical relevance of Parkinson’s disease (PD) is undisputed. PD occurs predominantly in the population over the age of 60 years with a global prevalence of around 1% increasing in tendency due to the aging population [2]. Aggregation of α-synuclein in the form of Lewy bodies promotes selective and progressive neuronal death leading to cardinal motor symptoms and a wide range of non-motor symptoms such as dysautonomia, constipation, and neuropsychiatric symptoms [3]. Polyneuropathy (PNP) is a pathologic condition of the peripheral nervous system affecting the sensory, motor, and autonomic domains [4]. Depending on the functional domain, PNP manifests with sensory symptoms such as numbness, paresthesia, pain, and motor weakness. In our study context, we refer to the impairment of the myelinated large nerve fibers. A higher coincidence of PNP has been observed in PD [5–9]. Nerve conduction studies revealed that in the majority of PD cases, PNP is distal, symmetric, axonal, and predominantly sensory [10]. As a result of PNP comorbidity, PD symptoms such as gait or sensory disturbances could be further compromised [11, 12]. The question of whether PNP occurrence is influenced by extrinsic factors as implied by data on levodopa utilization or is intrinsic to PD itself by α-synuclein pathology in peripheral nerves is still under debate [10, 13]. In this prospective study, we analyze the electrophysiological course of PNP in PD patients over a follow-up period of 2 years. Taking into account a variety of clinical and laboratory assessments, we evaluate the relevance of PNP in PD as a possible marker of disease progression.

## Methods

### Inclusion of patients

We extended the prospective monocentric “Parkinson Nerve Study” cohort at St. Josef-Hospital in Bochum, Germany, which had been established for our first observational study [5]. This study was registered in the German clinical trials registry (DRKS-ID: DRKS00020752) and approved by the Ethics Committee of the Medical Faculty of Ruhr University Bochum (Reg. No. 18-6360, date of approval 12.09.2018).

**Fig. 1:**
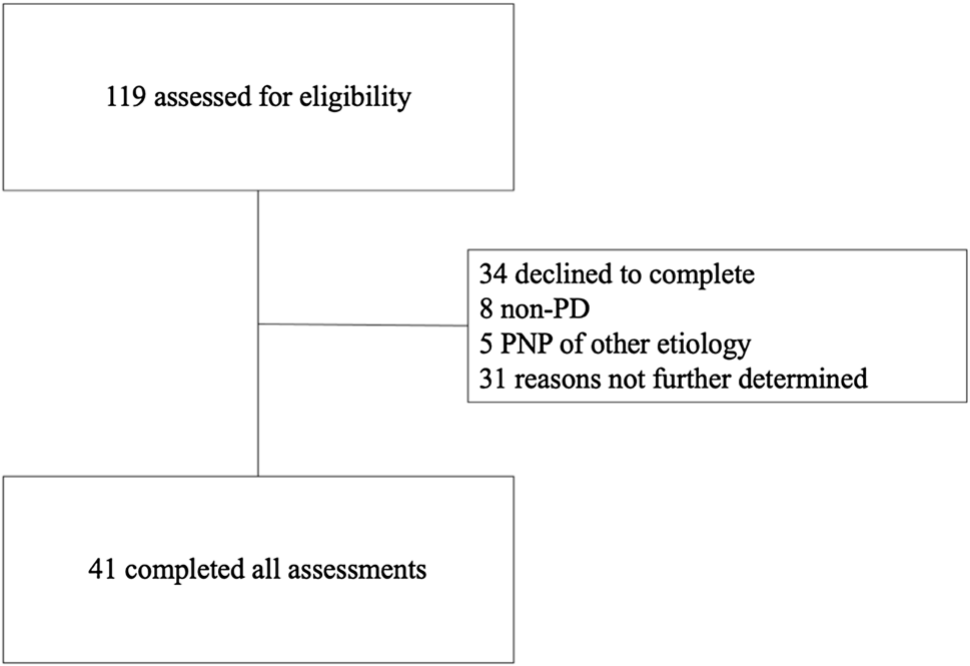
Study flow diagram

All patients participating in our study were seen by neurologists specialized in movement disorders. For eligibility, patients had to fulfill the criteria by the United Kingdom Parkinson’s Society Brain Bank [14] as well as those by the Movement Disorder Society for PD [15]. Patients with known causes of PNP such as diabetes or alcohol abuse, severe depression, or dementia were excluded from the study. All patients agreed to participate in the form of written informed consent. One hundred nineteen PD patients were enrolled between October 2018 and September 2021. Out of these patients, 41 participants could be recruited for follow-up after 2 years between September 2020 and August 2023. Others could not be followed-up due to various reasons (Fig. 1).

### Clinical evaluation

Medical history and sociodemographic data of all participants were assessed. Moreover, a detailed clinical examination as well as the acquisition of relevant clinical scores were carried out as part of the study. Clinical examinations that we applied in this study in order to evaluate the severity of motor and non-motor PD symptoms include Hoehn and Yahr Scale (H&Y; [16]) MDS-Unified Parkinson’s Disease Rating Scale (MDS-UPDRS) Part I, II and III [17], Non-Motor Symptoms Questionnaire (NMSQ; [18]) and Montreal Cognitive Assessment Test for Dementia (MoCA; [19]). Subitem 10 (unexplained pains) and subitem 21 (falling) of NMSQ were separately analyzed to evaluate PNP-typical symptoms. Subitem 2.12 (walking and balance) und 3.12 (postural stability) of MDS-UPDRS were summed up to focus on balance deficits and falls. Neuropathy Symptom Score (NSS; [20]) and Parkinson’s Disease Questionnaire (PDQ-39; [21]) were used to assess the subjective burden of PNP symptoms. For sub-analysis, we applied Stebbins’ categorization of clinical PD phenotypes into postural instability/gait difficulty (PIGD), tremor dominant (TD), and indeterminate subgroup [22].

### Assessment of laboratory values

To evaluate other causes of PNP and the influence of levodopa medication, our patients received blood sampling and urine analysis which were checked for abnormalities concerning blood cell count, HbA1c, liver enzymes, urea, electrolytes, creatinine, thyroid stimulating hormone, vitamin B12, B1, B6, methylmalonic acid, folic acid, homocysteine, holotranscobalamin and serum protein electrophoresis/immunfixation.

### Nerve conduction studies

Nerve conduction studies (NCS) are considered the gold standard method for objective and reliable evaluation of large nerve function [23]. We performed NCS on the peripheral nerves using a Medtronic four-channel electroneurography device (Medtronic, Meerbusch, Germany). Our protocol included an examination of motor amplitudes of the tibial, fibular, median and ulnar nerve and sensory studies of the fibular, radial, median, and ulnar nerve. Whenever possible, nerve conduction studies were performed bilaterally. The lower nerve amplitude was selected for early PNP detection. PNP was diagnosed based on the electrophysiological criteria by Stöhr et al., 2014 [24]. PNP severity was classified into mild, moderate, and severe. Mild sensory PNP was defined as a reduction of sensory nerve action potential (sNAP) < 3.8 µV of the sural nerve. Patients presenting with reduced sural sNAP as well as reduced compound muscle action potential (cMAP) of the tibial nerve < 5 mV were considered to be moderately affected. A severe, sensorimotor PNP was set if additionally, a reduction of median cMAP < 5 mV or median sNAP < 6.9 µV was present.

### Statistical analysis

SPSS version 28.0 (IBM Deutschland GmBH, Ehningen, Germany) was used for statistical analysis and graphics. The Shapiro–Wilk test was applied to assess normal distribution. In the case of a normal distribution, comparisons were made using the *t* test. Moreover, the Mann–Whitney *U* test and the Wilcoxon test were utilized for group comparisons concerning non-normally distributed data. To review co-variances between different variables, Spearman’s r was applied. Data were considered significantly different when p values fell below 0.05. To rule out age as a confounding factor of PNP, we performed partial correlation analysis with corrections for age.

## Results

### Clinical and PNP status at baseline

We included 41 PD patients for our longitudinal analysis as summarized in Table 1.

**Table 1:** Clinical and electrophysiological characteristics of the study population with and without PNP at baseline

	PNP( +) (*n* = 27)	PNP(–) (*n* = 14)	*p*
Age at examination (years)	66.33 ± 9.53 (27)	59.36 ± 6.63 (14)	0.019*
Female	8	9	
Disease duration (years)	5.67 ± 4.76 (27)	4.93 ± 3.73 (14)	0.658
Age at PD diagnosis (years)	60.67 ± 7.75 (27)	54.43 ± 7.27 (14)	0.017*
H&Y (median, IQR)	2 (IQR 1) (27)	2,25 (IQR 1) (14)	0.977
MDS-UPDRS I	10.73 ± 5.97 (26)	11.43 ± 6.16 (14)	0.898
MDS-UPDRS II	12.58 ± 9.04 (26)	11.43 ± 7.17 (14)	0.831
MDS-UPDRS III	32.59 ± 16.53 (27)	18.79 ± 7.96 (14)	< 0.001**
MDS-UPDRS II.12 + III.12	1.65 ± 2.10 (26)	1.14 ± 0.95 (14)	0.941
PDQ-39	22.38 ± 17.71 (26)	24.19 ± 15.49 (14)	0.533
NMSQ	9.04 ± 5.45 (26)	9.14 ± 4.72 (14)	0.921
NMSQ (item 10—yes)	4 (23)	2 (13)	
NMSQ (item 21—yes)	8 (24)	0 (14)	
NSS	5.46 ± 3.19 (26)	3.43 ± 2.93 (14)	0.065
MoCA	24.92 ± 3.63 (25)	23.71 ± 3.52 (14)	0.227
LED (mg)	610.15 ± 315.56 (27)	586.32 ± 391.47 (14)	0.834
Levodopa (mg)	329.63 ± 187.22 (27)	323.21 ± 207.19 (14)	0.921
Vitamin B12 (pg/ml)	421.08 ± 163.55 (26)	434.85 ± 105.54 (13)	0.753
Holotranscobalamin (pmol/l)	88.57 ± 34.89 (26)	82.84 ± 24.82 (14)	0.590
Folic acid (ng/ml)	13.03 ± 6.43 (26)	8.24 ± 4.84 (13)	0.037*
Methylmalonic acid (nmol/l)	353.63 ± 293.93 (25)	236.02 ± 71.60 (14)	0.224
Homocysteine (µmol/l)	19.07 ± 7.83 (20)	16.77 ± 5.70 (13)	0.507
Sural nerve (µV)	0.69 ± 1.21 (27)	6.92 ± 1.84 (14)	< 0.001**
Tibial nerve (mV)	5.35 ± 3.79 (27)	10.90 ± 3.35 (14)	< 0.001**
Median motor nerve (mV)	6.40 ± 1.57 (25)	6.27 ± 2.39 (13)	0.834
Median sensory nerve (µV)	7.28 ± 5.53 (24)	10.72 ± 7.59 (13)	0.105
Fibular motor nerve (mV)	2.53 ± 2.36 (11)	2.47 ± 1.98 (6)	0.958
Fibular sensory nerve (µV)	1.28 ± 2.63 (11)	3.08 ± 2.46 (6)	0.091
Radial nerve (µV)	5.53 ± 1.72 (10)	7.80 ± 4.71 (6)	0.301
Ulnar motor nerve (mV)	7.88 ± 1.31 (12)	8.54 ± 2.80 (7)	0.569
Ulnar sensory nerve (µV)	6.06 ± 1.49 (12)	5.70 ± 1.58 (7)	0.627

*PNP(+)* PD patients with PNP, *PNP(–)* denotes PD patients without PNP. *IQR* interquartile ratio, *H&Y* Hoehn and Yahr Scale, *MDS-UPDRS* Movement Disorder Society Unified Parkinson’s Disease Rating Scale, *NMSQ* Non-Motor Symptom Questionnaire, *NSS* Neuropathy Symptom Score, *MoCA* Montreal Cognitive Assessment, *LED* levodopa equivalence dose

*p < 0.05; **p < 0.01. Clinical scores: mean values ± standard deviation (SD) are presented. H&Y scale: median value and IQR are presented

PD patients with electrophysiological signs of PNP were significantly older at examination (*p* = 0.019) and at PD diagnosis (*p* = 0.017) compared to those without PNP (Table 1). Notably, MDS-UPDRS III was significantly higher in the PNP-positive subgroup (*p* < 0.001, Table 1). MDS-UPDRS II.12 and III.12 regarding balance and falls showed no difference between subgroups. NSS did not differ between PD patients with and without PNP. Only few patients reported unexplained pain (NMSQ item 10). Falls (NMSQ item 21) were only present in the PNP-positive subgroup. No difference could be found in terms of levodopa equivalence as well as isolated levodopa dosage. Regarding laboratory assessments, a higher folate level was detected in the PD-PNP subgroup (*p* = 0.037, Table 1) independent of folic acid intake (*r* = 0.186, *p* = 0.284).

**Fig. 2:**
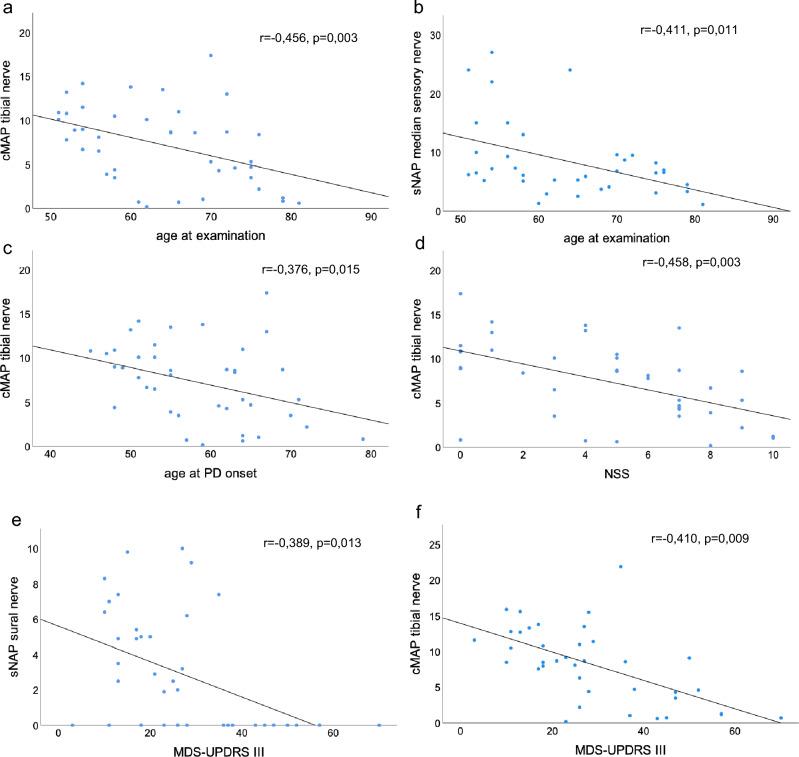
Correlations at baseline. **a** Amplitude of tibial nerve in relation to age at examination; **b** amplitude of median nerve (sensory) in relation to age at examination; **c** amplitude of tibial nerve in relation to age at PD onset; **d** amplitude of tibial nerve in relation to NSS; **e** amplitude of sural nerve in relation to MDS-UPDRS III; **f** amplitude of tibial nerve in relation to MDS-UPDRS III

**Fig. 3:**
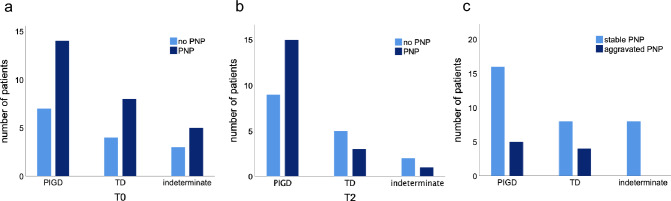
Distribution and longitudinal evolution of PNP in Stebbins’ phenotypes. Patients affected by PNP vs. patients not affected by PNP in Stebbins’ phenotypes at T0 (**a**) and T2 (**b**); **c** patients with stable vs. aggravated PNP in Stebbins’ phenotypes. *PIGD* postural instability and gait difficulties, *TD* tremor dominant

In total, 65.85% of our PD patients showed signs of an at least mild PNP at baseline. Reduced sural and tibial nerve amplitudes distinguished PNP-positive from the PNP-negative subgroup significantly (*p* < 0.001, Table 1). According to our PNP categorization, 31.71% showed mild sensory, 14.63% moderate sensorimotor, and 19.51% severe sensorimotor PNP. Tibial cMAP (*r* = – 0.456, *p* = 0.003, Fig. 2a) and median sNAP (*r* = – 0.411, *p* = 0.011, Fig. 2b) correlated inversely with age at examination. Moreover, tibial cMAP correlated inversely with age at PD onset (*r* = – 0.376, p = 0.015; Fig. 2c) as well as with NSS scores (*r* = – 0.458, *p* = 0.003 after correction for age; Fig. 2d). Independent of age, lower sural and tibial nerve amplitudes correlated with higher MDS-UPDRS III scores (sural nerve *r* = – 0.389, *p* = 0.013; Fig. 2e; tibial nerve *r* = – 0.410, *p* = 0.009; Fig. 2f). Regarding Stebbins’ phenotype distribution, 14 out of 21 PIGD-PD and 8 out of 12 TD-PD patients (66.67% respectively) as well as 5 out of 8 patients of the indeterminate subgroup (62.50%) displayed electrophysiological signs of PNP (Fig. 3a).

### Follow-up evaluation

#### Change of clinical data

Over the course of 2 years, we observed a significant worsening of disease severity according to Hoehn and Yahr scale (*p* = 0.004) whereas MDS-UPDRS III did not change significantly (Table 2a). However, MDS-UPDRS II.12 and III.12 regarding balance and falls increased significantly in the total PD cohort (*p* = 0.036, Table 2a) as well as in the PNP-positive subgroup (*p* = 0.012, Table 2b). NMSQ increased (NMSQ p = 0.028, Table 2a) and the PNP-specific subitems 10 and 12 also showed an increasing trend. Levodopa dosage increased, whereas homocysteine levels decreased significantly in the total PD cohort (LED/levodopa *p* < 0.001; homocysteine *p* = 0.045, Table 2a). Patients with electroneurographical features of PNP at baseline showed a deterioration of the Hoehn and Yahr scale (*p* = 0.004) and lower MoCA scores (*p* = 0.009) at T2, as outlined in Table 2b.

**Table 2:** Longitudinal evaluation of clinical and electrophysiological parameters

	T0 total (*n* = 41)	T2 total (*n* = 41)	*p*	T0 PNP( +) (*n* = 27)	T2 PNP( +) (*n* = 27)	*p*
H&Y (median, IQR)	2 (IQR 1.0)	2,5 (IQR 1.0)	0.004*	2.0 (IQR 1.0)	3.0 (IQR 1.0)	0.004*
MDS-UPDRS I	11.50 ± 5.93	11.78 ± 6.10	0.730	11.48 ± 5.79	11.70 ± 6.31	0.843
MDS-UPDRS II	12.18 ± 8.36	13.45 ± 9.04	0.925	12.58 ± 9.04	14.44 ± 10.16	0.552
MDS-UPDRS III	27.70 ± 15,72	28.18 ± 15,14	0.799	32,59 ± 16.53	33,67 ± 14.54	0.571
MDS-UPDRS II.12 + III.12	1,0.48 ± 1,0.78	2,0.13 ± 2,0.17	0,0.036*	1,0.65 ± 2,0.10	2,0.59 ± 2,0.34	0,0.012*
PDQ-39	23.72 ± 16.92	25.20 ± 18.21	0.562	22.38 ± 17.71	26.49 ± 20.68	0.187
NMSQ	9.10 ± 5.21	11.00 ± 5.26	0.028*	9.04 ± 5.45	10.54 ± 5.31	0.141
NMSQ (item 10—yes)	6 (36)	20 (39)		4 (23)	12 (27)	
NMSQ (item 21—yes)	8 (38)	15 (40)		8 (24)	11 (27)	
NSS	4.87 ± 3.16	5.05 ± 3.15	0.774	5.46 ± 3.19	5.58 ± 2.86	0.986
MoCA	24.54 ± 3.52	23.68 ± 4.03	0.089	24.79 ± 3.65	23.08 ± 4.38	0.009*
LED (mg)	602.01 ± 338.62	822.89 ± 357.18	< 0.001**	610.15 ± 315.56	885.07 ± 380.58	< 0.001**
Levodopa (mg)	327.44 ± 191.69	491.46 ± 233.09	< 0.001**	329.63 ± 187.22	532.41 ± 267.09	< 0.001**
Vitamin B12 (pg/ml)	433.12 ± 150.11	467.22 ± 165.14	0.270	428.44 ± 162.47	479.06 ± 171.92	0.192
Holotranscobalamin (pmol/l)	86.33 ± 32.60	91.36 ± 34.50	0.430	90.80 ± 34.62	96.53 ± 34.82	0.478
Folic acid (ng/ml)	12.06 ± 6.33	12.23 ± 6.50	0.567	13.41 ± 6.27	12.65 ± 6.42	0.191
Methylmalonic acid (nmol/l)	279.59 ± 138.48	280.82 ± 147.96	0.758	297.07 ± 155.24	289.34 ± 166.01	0.808
Homocysteine (µmol/l)	18.66 ± 6.77	15.97 ± 5.48	0.045*	19.40 ± 7.90	16.08 ± 6.51	0.059
Sural nerve (µV)	2.81 ± 3.32	3.69 ± 3.26	0.058	0.69 ± 1.21	2.65 ± 2.77	< 0.001**
Tibial nerve (mV)	7.24 ± 4.48	6.21 ± 4.02	0.004*	5.35 ± 3.79	4.61 ± 3.68	0.025*
Median sensory nerve (µV)	8.85 ± 6.63	4.78 ± 2.93	0.002*	7.43 ± 5.71	4.07 ± 2.19	0.011*
Fibular motor nerve (mV)	2.52 ± 2.28	2.44 ± 2.30	0.724	2.45 ± 2.50	2.34 ± 2.54	0.799
Fibular sensory nerve (µV)	1.61 ± 2.11	1.23 ± 3.34	0.753	0.81 ± 1.41	0.00 ± 0.00	0.180
Radial nerve (µV)	6.53 ± 3.78	6.58 ± 5.73	0.477	4.91 ± 1.50	2.29 ± 1.63	0.046*
Ulnar motor nerve (mV)	8.26 ± 2.11	7.39 ± 1.69	0.224	7.82 ± 1.45	7.89 ± 1.77	0.678
Ulnar sensory nerve (µV)	5.99 ± 1.65	5.01 ± 4.02	0.392	6.30 ± 1.67	3.09 ± 1.93	0.006*

^a^Evaluation of the total cohort at T0 and T2

^b^Evaluation of PNP-positive PD patients (T0) at T0 and T2

Clinical scores: mean values ± SD are presented. H&Y scale: median value and IQR are presented. NCS: mean amplitudes ± SD are presented. H&Y: median and IQR are presented. *p < 0.05, **p < 0.01

#### Change of PNP parameters

**Fig. 4:**
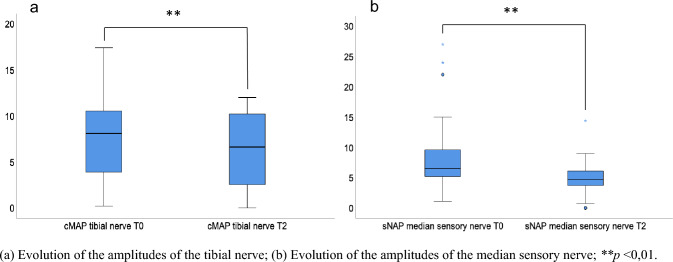
Longitudinal course of the tibial and median sensory nerve. **a** Evolution of the amplitudes of the tibial nerve; **b** evolution of the amplitudes of the median sensory nerve; **p < 0.01

In the nerve conduction analysis, median sNAP (p = 0.002) and tibial cMAP (*p* = 0.004) were shown to be significantly reduced at follow-up (Table 2a, Fig. 4a, b). Median sNAP decreased by 45%, tibial cMAP decreased by 14.2% in our total PD cohort. Comparing PNP-positive PD subgroups at T0 vs. T2, amplitudes of the tibial (*p* = 0.025, Fig. 4a), median sensory (*p* = 0.011), radial sensory (*p* = 0.046), and ulnar sensory (*p* = 0.006) nerve were significantly reduced, whereas the sural sNAP was increased (*p* < 0.001; Table 2b). Overall, 21.95% of the patients presented with either a deterioration of an established PNP into a more affected PNP category or developed electrophysiological signs of PNP for the first time. 78.05% of the patients were stable in PNP, 26.83% remained without PNP (Table S1). Patients who did not develop PNP exhibited lower UPDRS III (*p* = 0.002) and NSS scores (*p* = 0.023; Table S1), and were of younger age (*p* = 0.035). Comparing PD patients with stable versus aggravated PNP, NSS (*p* = 0.029) and vitamin B12 levels (*p* = 0.015) were higher in the aggravated subgroup (Table S2). On individual nerve level, a PNP deterioration was defined as a reduction of the nerve amplitude of at least 2 mV/µV. Sural sNAP worsened in 12.20%, tibial cMAP in 24.39%, and median cMAP in 25.71% of cases. A deterioration of the median sNAP was detected in 57.58% of cases. Referring to Stebbins’ phenotypes, 15 out of 24 patients of the PIGD subtype (62.50%), 3 out of 8 patients of the TD subtype (37.50%), and 1 out of 3 patients of the indeterminate subtype (33.33%) showed nerve conduction abnormalities at 2 years of follow-up (Fig. 3b). In sum, PNP status in the indeterminate group remained stable while a PNP progression was observed in 23.81% of PIGD and 33.33% of TD cases (Fig. 3c).

**Fig. 5:**
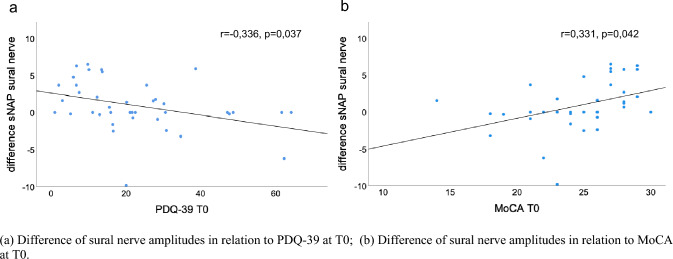
Correlation analysis in the longitudinal course. **a** Difference of sural nerve amplitudes in relation to PDQ-39 at T0; **b** difference of sural nerve amplitudes in relation to MoCA at T0

Correlation analysis revealed a significant association of the difference of the sural nerve amplitudes with PDQ-39 (*r* = – 0.336, *p* = 0.037; Fig. 5a) and MoCA scores (*r* = 0.331, *p* = 0.042; Fig. 5b) at T0 independent of patient age. Other nerve amplitudes in multiple combinations with clinical and laboratory parameters did not show a significant correlation.

## Discussion

Despite growing evidence of increased PNP prevalence in the PD population, longitudinal nerve conduction studies in PD patients are lacking. Therefore, we investigated the course of PNP in PD over 2 years to better understand the relationship between these entities.

Electrophysiological diagnosis of PNP could be established in 65.85% of our PD cohort at baseline. The higher PNP prevalence is in line with the findings of previous studies, although PNP rates vary greatly from 5% up to 69% [5, 25, 26] due to the heterogeneity of the study population and definition of PNP diagnosis. Some studies applied the American Academy of Neurology (AAN) PNP criteria requiring the combination of clinical and electrodiagnostic abnormalities [27] that could have lowered the overall PNP rate [6, 7, 25]. Whereas in our study, PNP diagnosis mainly relied on electrophysiological parameters, and selection of the lower amplitude of bilateral nerve measurements increased the sensitivity of PNP detection. In our study, we focused on the investigation of large fiber neuropathy. For this purpose, we chose NCS as an objective and reliable method to measure large nerve function. There are further specific neurophysiological tests such as quantitative sensory testing (QST) based on subjective perception thresholds and also integrating small nerve fiber evaluation [28]. Quantitative characterization of nerve fibers constitutes a growing field for evaluation of small fiber neuropathy [29]. Future studies should expand modalities of nerve assessment methods to provide a more integral understanding of peripheral nerve involvement in PD.

Age at examination and at PD diagnosis was higher in PD patients with PNP and correlated inversely with amplitudes of the tibial and median nerve. Patients who remained without PNP were also younger than patients with PNP in our study. These findings are intriguing since age has been discussed as an independent risk factor for the development of neuropathy. Ceravalo et al. reported that the risk of neuropathy increased by approximately 8% for each year of age [25]. Age-related nutritional deficiencies such as vitamin B12 status may partly influence the onset of neuropathy in PD [30]. Nevertheless, using age-matched controls, a higher PNP prevalence in PD has been confirmed in several studies [7, 25, 31, 32].

The majority of PNP cases at baseline showed length-dependently a distal predilection site for sensory and motor nerve impairment of the lower extremities (mild and moderate PNP), although 19.5% of PNP cases also involved the median nerve categorized as severe PNP. Using a different classification of neuropathy severity, Ramachandran et al. reported that out of 28 PD patients with PNP, 15 had mild axonal sensory neuropathy (below 2 SD sNAP), 8 had severe axonal sensory neuropathy (absent sNAP), and 5 had sensorimotor axonal neuropathy (below 2 SD cMAP) [33]. Another nerve conduction study found that the superficial fibular nerve (55.00%) and the sural nerve (50.00%) were most affected in PD patients [8]. These results support the general conception that PD-associated PNP is predominantly sensory and axonal [10], whereas in our cohort, motor amplitudes were also significantly affected.

At 2 years of follow-up, an overall PNP progression was observed in 21.95% of our PD cohort. This finding is intriguing since progression rates of idiopathic and vitamin B12 deficiency PNP have been reported to be minimal over 3 years [34]. Paradoxically, sural sNAP in our PD cohort increased over time. Technical issues such as electrical interference, excessive adipose tissue and edema in limbs could have led to variations in sNAP values [35, 36]. PNP progression affected the tibial nerve and nerves of the upper extremities. Of those, median sNAP showed the strongest amplitude reduction over 2 years and deterioration of sensory median nerve was most prevalent among our PD patients. An increased median nerve vulnerability has been discussed in PD patients. Yardimci et al. detected a demyelinating median neuropathy in 16.12% of PD cases that was bilateral in two-thirds of the patients [32]. Furthermore, sonography of the median nerve displayed an increased cross-sectional area in PD patients compared to controls [37]. Our findings, hence, advocate monitoring PNP progression of lower and upper extremities with particular consideration of the median nerve. NSS correlated with tibial nerve amplitude at baseline. Moreover, NSS was more elevated in the PD subgroup that developed PNP or suffered a PNP progression. Therefore, this scoring system could be a supportive tool to screen for PNP progression also associated with PD. Neuropathic pain constitutes a severe symptom burden requiring symptomatic relief. NMSQ subitem 10 addresses the important aspect of pain, although irrespective of cause, and should be considered for PNP evaluation in PD.

With regard to functional impact, reduced nerve amplitudes correlated with higher motor scores at baseline. Correlations between PNP and disease severity (Hoehn and Yahr, UPDRS) have been reported in previous studies [6, 7]. The deterioration of the Hoehn and Yahr stage was more pronounced in our PNP-positive PD cohort transitioning from 2.0 to 3.0 which marks the beginning of a postural instability and has high clinical relevance. Furthermore, focused analysis of balance and gait revealed a higher presence of falls in our PNP-positive subgroup as indicated by NMSQ subitem 21 and a deterioration of balance and gait (UPDRS II.12 + III.12) in all PD cases. Studies have shown that gait and balance disturbances in PD can be aggravated by PNP comorbidity [11, 38]. Beaulieu et al. reported that presence of PNP was significantly associated with more falls, shorter stride length, and slower gait speed, but no difference in the MDS-UPDRS motor examination scores [11]. Therefore, sub-analysis of motor and non-motor scores can help detect differences of PNP-relevant symptoms in PD. Corra et al. performed objective gait and balance assessment using wearable health-technology and demonstrated shorter stride length, slower gait speed, and smaller toe-off angles in PD patients with PNP comorbidity [38]. In our study, no correlation could be found between PNP progression and motor progression over the disease course. Decrease of sural nerve amplitude exhibited correlations with non-motor scores at baseline (PDQ-39, MoCA). Notably, Merola et al. suggested PNP as a marker of severe PD phenotype and showed that PNP is independently associated with cognitive decline, worse axial motor features, and worse non-motor symptoms [39]. However, findings regarding isolated sural nerve amplitude should be interpreted with caution for aforementioned reasons and more sensory nerve measurements should be taken into account to provide a broader perspective. In terms of phenotypical PD subtypes, our findings revealed a higher PNP prevalence in the TD and PIGD subgroup. Although the proportion of TD subgroup patients with PNP decreases after 2 years, the percentually strongest PNP aggravation was observed in the TD subgroup followed by PIGD subgroup. These trends indicate a possible impact of PNP on specific motor symptoms in PD.

As for large fiber neuropathy, levodopa treatment has been discussed as a risk factor for PNP development in PD [10, 13]. Baseline levodopa dosage did not differ between PD patients presenting with and without PNP. In our study, we referred to the daily levodopa dose, which was rather low with approximately 300 mg levodopa. Other studies comparing daily levodopa doses were also not able to detect a difference between PNP-positive and PNP-negative PD patients [38, 40]. In contrast, cumulative levodopa dosage and duration of levodopa exposure are suggested to impact PNP prevalence and severity [7, 25, 30]. Levodopa dosage increased over the course of 2 years. However, the difference of sural sNAP did not correlate with levodopa dosage in our study. Elevated levels of homocysteine and methylmalonic acid and vitamin B12 deficiency have been attributed to levodopa metabolism [25, 26, 41]. Despite an increase in levodopa dose, homocysteine levels in our PD cohort decreased. No correlation was found between sural sNAP and homocysteine levels, although an earlier study reported a significant inverse association between homocysteine levels and sNAP of the sural nerve [31]. Beyond extrinsic risk factors, an intrinsic cause of neurodegeneration of peripheral nerves should also be taken into consideration. Zhang et al. first verified the deposition of phosphorylated α-synuclein in sural nerve tissue explicitly in PD patients which may add to PNP pathology [42]. The expression of α-synuclein was attributed to Schwann cells supporting the hypothesis of a peripheral origin in peripheral nerve involvement in PD. In comparison to PD, notably fewer studies have tackled the question of peripheral nerve alterations in atypical Parkinsonian syndromes (APS) [43–46]. Our study group previously observed a high prevalence of subjective neuropathic symptom burden with electrophysiological PNP confirmation in 50% of patients with multiple system atrophy (MSA) and progressive supranuclear palsy (PSP) using the current NCS protocol [46]. Based on NCS, it is not possible to distinguish PD from non-idiopathic forms of parkinsonism. In skin biopsies, alpha-synuclein deposits were detected in MSA and PD patients only, but not in tauopathies or controls suggesting its potential role as a biomarker [47]. Evidence of phosphorylated α-synuclein accumulation in Schwann cells of MSA patients and tau pathology in cranial and spinal nerves of PSP patients indicates peripheral nerve involvement in APS [48, 49]. Further investigation is needed to determine distinct PNP characteristics and differences of PD and APS that may suggest biomarker potential.

A limiting factor of our monocentric study is the small number of patients that could be consistently followed over 2 years. Unfortunately, we lost a significant portion of patients to follow-up. One of the various reasons is due to the fact that parts of our follow-up visits took place during the Covid-19 pandemic, when restrictions of social contacts were required. Moreover, for patients with more severe disease activity, it was even more difficult to reappear to the visits which could have biased the composition of our PD cohort.

In conclusion, our findings reveal the high prevalence of PNP in PD patients that could add to the motor and sensory symptoms of PD. For the first time, we tracked PNP progression over the course of 2 years. Electrophysiological deterioration was detected in one-fifth of PNP cases including sensory nerves of the upper extremities what needs to be considered for monitoring. PNP progression parallels PD progression especially pronounced in TD and PIGD subgroups. Furthermore, correlation analysis of sural nerve amplitudes indicates that PNP could be a manifestation of non-motor symptoms. Therefore, longitudinal evaluation in a larger cohort and expansion of qualitative and quantitative nerve assessment tests will be necessary to further elucidate the involvement of peripheral neuropathy in PD. The investigation for putative etiologic correlates will additionally require the examination of biosamples such as skin and nerve biopsies.

## Supplementary Information

Below is the link to the electronic supplementary material.

Supplementary file1 (DOCX 25 KB)

## Data Availability

The authors confirm that the data supporting the findings of this study are available within the article and its supplementary materials.

## References

[1] Mhyre TR, Boyd JT, Hamill RW, Maguire-Zeiss KA (2012) Parkinson’s disease. Subcell Biochem 65:389–455. 10.1007/978-94-007-5416-4_16 23225012 10.1007/978-94-007-5416-4_16 PMC4372387

[2] Tysnes O-B, Storstein A (2017) Epidemiology of Parkinson’s disease. J Neural Transm (Vienna) 124:901–905. 10.1007/s00702-017-1686-y 28150045 10.1007/s00702-017-1686-y

[3] Sveinbjornsdottir S (2016) The clinical symptoms of Parkinson’s disease. J Neurochem 139(Suppl 1):318–324. 10.1111/jnc.13691 27401947 10.1111/jnc.13691

[4] Sommer C, Geber C, Young P, Forst R, Birklein F, Schoser B (2018) Polyneuropathies. Dtsch Arztebl Int 115:83–90. 10.3238/arztebl.2018.083 29478436 10.3238/arztebl.2018.083 PMC5832891

[5] Kühn E, Averdunk P, Huckemann S, Müller K, Biesalski A-S, zum Hof Berge F, Motte J, Fisse AL, Schneider-Gold C, Gold R et al (2020) Correlates of polyneuropathy in Parkinson’s disease. Ann Clin Transl Neurol 7:1898–1907. 10.1002/acn3.51182 32940017 10.1002/acn3.51182 PMC7545593

[6] Mathukumalli NL, Kandadai MR, Shaik JA, Kanikannan MA, Borgohain R (2020) Serum B12, Homocysteine Levels, and their Effect on Peripheral Neuropathy in Parkinson’s Disease: Indian Cohort. Ann Indian Acad Neurol 23:48–53. 10.4103/aian.AIAN_478_18 32055122 10.4103/aian.AIAN_478_18 PMC7001434

[7] Toth C, Breithaupt K, Ge S, Duan Y, Terris JM, Thiessen A, Wiebe S, Zochodne DW, Suchowersky O (2010) Levodopa, methylmalonic acid, and neuropathy in idiopathic Parkinson disease. Ann Neurol 68:28–36. 10.1002/ana.22021 20582991 10.1002/ana.22021

[8] Hernandez Fustes OJ, Hernandez Fustes OJ (2020) Sensory Neuropathy in Parkinson Disease: Electrodiagnostic Evaluation. Neurodiagn J 60:177–184. 10.1080/21646821.2020.1796414 33006509 10.1080/21646821.2020.1796414

[9] de Araújo DF, de Melo Neto AP, Oliveira ÍSC, Brito BS, de Araújo IT, Barros IS, Lima JWO, Horta WG, Gondim FdAA (2016) Small (autonomic) and large fiber neuropathy in Parkinson disease and parkinsonism. BMC Neurol 20:16. 10.1186/s12883-016-0667-3 10.1186/s12883-016-0667-3 PMC4988006 27530902

[10] Zis P, Grünewald RA, Chaudhuri RK, Hadjivassiliou M (2017) Peripheral neuropathy in idiopathic Parkinson’s disease: a systematic review. J Neurol Sci 378:204–209. 10.1016/j.jns.2017.05.023 28566165 10.1016/j.jns.2017.05.023

[11] Beaulieu ML, Müller ML, Bohnen NI (2018) Peripheral neuropathy is associated with more frequent falls in Parkinson’s disease. Parkinsonism Relat Disord 54:46–50. 10.1016/j.parkreldis.2018.04.006 29625874 10.1016/j.parkreldis.2018.04.006 PMC6163065

[12] Tai Y-C, Lin C-H (2019) An overview of pain in Parkinson’s disease. Clin Park Relat Disord 2:1–8. 10.1016/j.prdoa.2019.11.004 34316612 10.1016/j.prdoa.2019.11.004 PMC8302194

[13] Paul DA, Qureshi ARM, Rana AQ (2020) Peripheral neuropathy in Parkinson’s disease. Neurol Sci 41:2691–2701. 10.1007/s10072-020-04407-4 32358706 10.1007/s10072-020-04407-4

[14] Gibb WR, Lees AJ (1988) The relevance of the Lewy body to the pathogenesis of idiopathic Parkinson’s disease. J Neurol Neurosurg Psychiatry 51:745–752 2841426 10.1136/jnnp.51.6.745 PMC1033142

[15] Postuma RB, Berg D, Stern M, Poewe W, Olanow CW, Oertel W, Obeso J, Marek K, Litvan I, Lang AE et al (2015) MDS clinical diagnostic criteria for Parkinson’s disease. Mov Disord 30:1591–1601. 10.1002/mds.26424 26474316 10.1002/mds.26424

[16] Hoehn MM, Yahr MD (1967) Parkinsonism: onset, progression and mortality. Neurology 17:427–442. 10.1212/wnl.17.5.427 6067254 10.1212/wnl.17.5.427

[17] Goetz CG, Tilley BC, Shaftman SR, Stebbins GT, Fahn S, Martinez-Martin P, Poewe W, Sampaio C, Stern MB, Dodel R et al (2008) Movement Disorder Society-sponsored revision of the Unified Parkinson’s Disease Rating Scale (MDS-UPDRS): scale presentation and clinimetric testing results. Mov Disord 23:2129–2170. 10.1002/mds.22340 19025984 10.1002/mds.22340

[18] Chaudhuri KR, Martinez-Martin P, Schapira AHV, Stocchi F, Sethi K, Odin P, Brown RG, Koller W, Barone P, MacPhee G et al (2006) International multicenter pilot study of the first comprehensive self-completed nonmotor symptoms questionnaire for Parkinson’s disease: the NMSQuest study. Mov Disord 21:916–923. 10.1002/mds.20844 16547944 10.1002/mds.20844

[19] Nasreddine ZS, Phillips NA, Bédirian V, Charbonneau S, Whitehead V, Collin I, Cummings JL, Chertkow H (2005) The Montreal Cognitive Assessment, MoCA: a brief screening tool for mild cognitive impairment. J Am Geriatr Soc 53:695–699. 10.1111/j.1532-5415.2005.53221.x 15817019 10.1111/j.1532-5415.2005.53221.x

[20] Dyck PJ, Sherman WR, Hallcher LM, Service FJ, O’Brien PC, Grina LA, Palumbo PJ, Swanson CJ (1980) Human diabetic endoneurial sorbitol, fructose, and myo-inositol related to sural nerve morphometry. Ann Neurol 8:590–596. 10.1002/ana.410080608 7212646 10.1002/ana.410080608

[21] Jenkinson C, Fitzpatrick R, Peto V, Greenhall R, Hyman N (1997) The Parkinson’s Disease Questionnaire (PDQ-39): development and validation of a Parkinson’s disease summary index score. Age Ageing 26:353–357. 10.1093/ageing/26.5.353 9351479 10.1093/ageing/26.5.353

[22] Stebbins GT, Goetz CG, Burn DJ, Jankovic J, Khoo TK, Tilley BC (2013) How to identify tremor dominant and postural instability/gait difficulty groups with the movement disorder society unified Parkinson’s disease rating scale: comparison with the unified Parkinson’s disease rating scale. Mov Disord 28:668–670. 10.1002/mds.25383 23408503 10.1002/mds.25383

[23] Carmichael J, Fadavi H, Ishibashi F, Shore AC, Tavakoli M (2021) Advances in screening, early diagnosis and accurate staging of diabetic neuropathy. Front Endocrinol (Lausanne) 12:671257. 10.3389/fendo.2021.671257 34122344 10.3389/fendo.2021.671257 PMC8188984

[24] Stöhr M, Pfister R, Reilich P (2022) Klinische Elektromyographie und Neurographie: Lehrbuch und Atlas, 7 erweiterte und überarbeiteteAuflage. Kohlhammer Verlag, Stuttgart (**ISBN 978-3-17-035043-4**)

[25] Ceravolo R, Cossu G, Di Bandettini Poggio M, Santoro L, Barone P, Zibetti M, Frosini D, Nicoletti V, Manganelli F, Iodice R et al (2013) Neuropathy and levodopa in Parkinson’s disease: evidence from a multicenter study. Mov Disord 28:1391–1397. 10.1002/mds.25585 23836370 10.1002/mds.25585

[26] Toth C, Brown MS, Furtado S, Suchowersky O, Zochodne D (2008) Neuropathy as a potential complication of levodopa use in Parkinson’s disease. Mov Disord 23:1850–1859. 10.1002/mds.22137 18785232 10.1002/mds.22137

[27] England JD, Gronseth GS, Franklin G, Miller RG, Asbury AK, Carter GT, Cohen JA, Fisher MA, Howard JF, Kinsella LJ et al (2005) Distal symmetric polyneuropathy: a definition for clinical research: report of the American Academy of Neurology, the American Association of Electrodiagnostic Medicine, and the American Academy of Physical Medicine and Rehabilitation. Neurology 64:199–207. 10.1212/01.WNL.0000149522.32823.EA 15668414 10.1212/01.WNL.0000149522.32823.EA

[28] Mücke M, Cuhls H, Radbruch L, Baron R, Maier C, Tölle T, Treede R-D, Rolke R (2021) Quantitative sensorische Testung (QST). Schmerz 35:153–160. 10.1007/s00482-015-0093-2 26826097 10.1007/s00482-015-0093-2

[29] Gasparotti R, Padua L, Briani C, Lauria G (2017) New technologies for the assessment of neuropathies. Nat Rev Neurol 13:203–216. 10.1038/nrneurol.2017.31 28303912 10.1038/nrneurol.2017.31

[30] Rajabally YA, Martey J (2013) Levodopa, vitamins, ageing and the neuropathy of Parkinson’s disease. J Neurol 260:2844–2848. 10.1007/s00415-013-7079-8 23989342 10.1007/s00415-013-7079-8

[31] Müller T, Renger K, Kuhn W (2004) Levodopa-associated increase of homocysteine levels and sural axonal neurodegeneration. Arch Neurol 61:657–660. 10.1001/archneur.61.5.657 15148140 10.1001/archneur.61.5.657

[32] Yardimci N, Cemeroglu O, Ozturk E, Gürlü G, Şahin E, Bozkurt S, Cengiz T, Karali G, Cakirbay H, İlhan A (2016) Median and ulnar neuropathy assessment in Parkinson’s disease regarding symptom severity and asymmetry. Parkinsons Dis. 10.1155/2016/4958068 27843673 10.1155/2016/4958068 PMC5098091

[33] Ramachandran A, Jose J, Gafoor VA, Das S, Balaram N (2022) Prevalence and risk factors of peripheral neuropathy in Parkinson’s disease. Ann Indian Acad Neurol 25:1109–1115. 10.4103/aian.aian_669_22 36911466 10.4103/aian.aian_669_22 PMC9996500

[34] Sachedina S, Toth C (2013) Progression in idiopathic, diabetic, paraproteinemic, alcoholic, and B12 deficiency neuropathy. J Peripher Nerv Syst 18:247–255. 10.1111/jns5.12042 24028193 10.1111/jns5.12042

[35] Sorenson EJ (2021) Sensory nerve conduction studies and sensory nerve action potentials. In: Rubin DI (ed) Clinical neurophysiology. Oxford University Press, New York (**325-C18.P131, ISBN 0190067853**)

[36] Sreenivasan A, Mansukhani KA, Sharma A, Balakrishnan L (2016) Sural sensory nerve action potential: a study in healthy Indian subjects. Ann Indian Acad Neurol 19:312–317. 10.4103/0972-2327.186786 27570380 10.4103/0972-2327.186786 PMC4980951

[37] Atwan H, Ch MBB, Abdelaziz A, Kassem HA, Eltobgy MA, Gamal M, Sleem A, Ebaid NY (2024) Median nerve ultrasonography in Parkinson’s disease: a systematic review and meta-analysis. Int J Neurosci. 10.1080/00207454.2024.2327407 38497467 10.1080/00207454.2024.2327407

[38] Corrà MF, Vila-Chã N, Sardoeira A, Hansen C, Sousa AP, Reis I, Sambayeta F, Damásio J, Calejo M, Schicketmueller A et al (2023) Peripheral neuropathy in Parkinson’s disease: prevalence and functional impact on gait and balance. Brain 146:225–236. 10.1093/brain/awac026 35088837 10.1093/brain/awac026 PMC9825570

[39] Merola A, Rosso M, Romagnolo A, Comi C, Fasano A, Zibetti M, Lopez-Castellanos JR, Cocito D, Lopiano L, Espay AJ (2017) Peripheral neuropathy as marker of severe Parkinson’s disease phenotype. Mov Disord 32:1256–1258. 10.1002/mds.27025 28582598 10.1002/mds.27025

[40] Shahrizaila N, Mahamad UA, Yap A-C, Choo Y-M, Marras C, Lim S-Y (2013) Is chronic levodopa therapy associated with distal symmetric polyneuropathy in Parkinson’s disease? Parkinsonism Relat Disord 19:391–393. 10.1016/j.parkreldis.2012.08.002 23146348 10.1016/j.parkreldis.2012.08.002

[41] Rajabally YA, Martey J (2011) Neuropathy in Parkinson disease: prevalence and determinants. Neurology 77:1947–1950. 10.1212/WNL.0b013e31823a0ee4 22049200 10.1212/WNL.0b013e31823a0ee4

[42] Zhang H, Zhu L, Sun L, Zhi Y, Ding J, Yuan Y-S, Shen F-F, Li X, Ji P, Wang Z et al (2019) Phosphorylated α-synuclein deposits in sural nerve deriving from Schwann cells: A biomarker for Parkinson’s disease. Parkinsonism Relat Disord 60:57–63. 10.1016/j.parkreldis.2018.10.003 30297212 10.1016/j.parkreldis.2018.10.003

[43] Gawel M, Jamrozik Z, Szmidt-Salkowska E, Slawek J, Gawel D, Rowińska-Marcińska K, Kaminska A (2013) Electrophysiological features of lower motor neuron involvement in progressive supranuclear palsy. J Neurol Sci 324:136–139. 10.1016/j.jns.2012.10.023 23146616 10.1016/j.jns.2012.10.023

[44] Gawel M, Jamrozik Z, Szmidt-Salkowska E, Slawek J, Rowinska-Marcinska K (2012) Is peripheral neuron degeneration involved in multiple system atrophy? A clinical and electrophysiological study. J Neurol Sci 319:81–85. 10.1016/j.jns.2012.05.011 22647584 10.1016/j.jns.2012.05.011

[45] Abele M, Schulz JB, Bürk K, Topka H, Dichgans J, Klockgether T (2000) Nerve conduction studies in multiple system atrophy. Eur Neurol 43:221–223. 10.1159/000008179 10828652 10.1159/000008179

[46] Rohmann R, Kühn E, Scherbaum R, Hilker L, Kools S, Scholz L, Müller K, Huckemann S, Schneider-Gold C, Gold R et al (2021) Prevalence and Characteristics of Polyneuropathy in Atypical Parkinsonian Syndromes: An Explorative Study. Brain Sci. 10.3390/brainsci11070879 34209067 10.3390/brainsci11070879 PMC8301815

[47] Doppler K, Weis J, Karl K, Ebert S, Ebentheuer J, Trenkwalder C, Klebe S, Volkmann J, Sommer C (2015) Distinctive distribution of phospho-alpha-synuclein in dermal nerves in multiple system atrophy. Mov Disord 30:1688–1692. 10.1002/mds.26293 26175301 10.1002/mds.26293

[48] Tanaka H, Martinez-Valbuena I, Forrest SL, Couto B, Reyes NG, Morales-Rivero A, Lee S, Li J, Karakani AM, Tang-Wai DF et al (2024) Distinct involvement of the cranial and spinal nerves in progressive supranuclear palsy. Brain 147:1399–1411. 10.1093/brain/awad381 37972275 10.1093/brain/awad381 PMC10994524

[49] Nakamura K, Mori F, Kon T, Tanji K, Miki Y, Tomiyama M, Kurotaki H, Toyoshima Y, Kakita A, Takahashi H et al (2015) Filamentous aggregations of phosphorylated α-synuclein in Schwann cells (Schwann cell cytoplasmic inclusions) in multiple system atrophy. Acta Neuropathol Commun 3:29. 10.1186/s40478-015-0208-0 25990096 10.1186/s40478-015-0208-0 PMC4438578

