# Supplementary material for: Longitudinal evaluation of polyneuropathy in Parkinson’s disease
Source: J Neurol. 2024 Jul 26;271(9):6136–46. doi: 10.1007/s00415-024-12579-8 (PMC11377511; doi:10.1007/s00415-024-12579-8)
Supplement: Supplementary file 1 — Supplementary file1 (DOCX 25 KB) [file 415_2024_12579_MOESM1_ESM.docx]

**Table S1.**

*Comparison of patients remaining without PNP versus patients with PNP at T0/T2*

|  | no PNP at T0/T2 (n=11) | PNP at T0/T2 (n=30) | p |
| --- | --- | --- | --- |
|  |  |  |  |
| age (years) | 58.82 ± 6.15 | 65.83 ± 9.48 | 0.035* |
| disease duration (years) | 4.73 ± 3.82 | 5.67 ± 4.63 | 0.505 |
| age at PD diagnosis (years) | 54.09 ± 6.94 | 60.17 ± 7.94 | 0.031* |
| H&Y (median, IQR) | 2.0 (IQR 1.0) | 2.25 (IQR 1.0) | 0.807 |
| MDS-UPDRS III | 18.45 ± 8.49 | 31.33 ± 16.22 | 0.002* |
| MDS-UPDRS I | 10.27 ± 3.74 | 11.24 ± 6.66 | 0.653 |
| PDQ-39 | 21.79 ± 12.34 | 23.48 ± 18.36 | 0.844 |
| NMSQ | 8.73 ± 2.94 | 9.21 ± 5.81 | 0.820 |
| NSS | 2.91 ± 2.63 | 5.45 ± 3.18 | 0.023* |
| MoCA | 23.91 ± 3.96 | 24.71 ± 3.48 | 0.616 |
|  |  |  |  |
| LED (mg) | 616.50 ± 439.25 | 596.70 ± 302.52 | 0.965 |
| Levodopa (mg) | 311.36 ± 233.28 | 333.33 ± 178.27 | 0.750 |
|  |  |  |  |
| vitamin B12 (pg/ml) | 424.90 ± 92.46 | 425.93 ± 161.14 | 0.981 |
| holotranscobalamin (pmol/l) | 82.31 ± 24.52 | 88.18 ± 34.05 | 0.607 |
| folic acid (ng/ml) | 8.59 ± 5.51 | 12.42 ± 6.36 | 0.095 |
| methylmalonic acid (nmol/l) | 235.88 ± 65.48 | 341.08 ± 281.13 | 0.268 |
| homocysteine (µmol/l) | 17.07 ± 6.47 | 18.64 ± 7.39 | 0.724 |
|  |  |  |  |
| sural nerve (µV) | 6.91 ± 1.76 | 1.31 ± 2.33 | <0.001** |
| tibial nerve (mV) | 11.68 ± 2.73 | 5.61 ± 3.86 | <0.001** |
| median motor nerve (mV) | 7.04 ± 1.94 | 6.11 ± 1.80 | 0.179 |
| median sensory nerve (µV) | 11.65 ± 8.49 | 7.32 ± 5.22 | 0.161 |
| fibular motor nerve (mV) | 2.28 ± 1.29 | 2.58 ± 2.41 | 0.820 |
| fibular sensory nerve (µV) | 3.75 ± 2.51 | 1.35 ± 2.51 | 0.066 |
| radial nerve (µV) | 5.63 ± 2.45 | 6.64 ± 3.51 | 0.396 |
| ulnar motor nerve (mV) | 7.72 ± 2.86 | 8.26 ± 1.62 | 0.703 |

| ulnar sensory nerve (µV) | 5.88 ± 1.80 | 5.94 ± 1.44 | 0.938 |
| --- | --- | --- | --- |

Clinical scores: mean values ± SD are presented. H&Y scale: median value and IQR are presented. NCS: mean amplitudes ± SD are presented. HuY: median and IQR are presented. **p* <0.05; ***p* <0.01.

|  | stable PNP (n=32) | aggravated PNP (n=9) | p |
| --- | --- | --- | --- |
|  |  |  |  |
| age (years) | 63,25 ± 9,48 | 66,44 ± 8,10 | 0,284 |
| disease duration (years) | 5,28 ± 4,63 | 5,89 ± 3,66 | 0,428 |
| age at PD diagnosis (years) | 57,97 ± 8,09 | 60,56 ± 8,14 | 0,403 |
| H&Y (median, IQR) | 2.0 (IQR 1.0) | 2.5 (IQR 1.5) | 0,948 |
| MDS-UPDRS III | 27,00 ± 14,93 | 31,00 ± 18,23 | 0,518 |
| MDS-UPDRS I | 10,45 ± 5,18 | 12,78 ± 8,27 | 0,708 |
| PDQ-39 | 21,88 ± 14,87 | 26,92 ± 22,85 | 0,662 |
| NMSQ | 8,61 ± 4,51 | 10,67 ± 7,02 | 0,637 |
| NSS | 4,25 ± 3,22 | 6,75 ± 2,43 | 0,029* |
| MoCA | 24,70 ± 3,79 | 23,78 ± 2,91 | 0,235 |
|  |  |  |  |
| LED (mg) | 627,61 ± 376,98 | 511,02 ± 103,36 | 0,128 |
| Levodopa (mg) | 314,84 ± 213,35 | 372,22 ± 66,67 | 0,198 |
|  |  |  |  |
| vitamin B12 (pg/ml) | 395,30 ± 129,53 | 526,89 ± 157,15 | 0,015* |
| holotranscobalamin (pmol/l) | 82,87 ± 30,36 | 99,28 ± 33,95 | 0,195 |
| folic acid (ng/ml) | 11,30 ± 6,45 | 11,88 ± 6,17 | 0,737 |
| methylmalonic acid (nmol/l) | 336,58 ± 269,10 | 227,52 ± 101,14 | 0,243 |
| homocysteine (µmol/l) | 18,55 ± 7,82 | 16,74 ± 2,80 | 0,982 |

**Table S2.**

*Comparison of patients with stable versus aggravated PNP*

Clinical scores: mean values ± SD are presented. H&Y scale: median value and IQR are presented. NCS: mean amplitudes ± SD are presented. HuY: median and IQR are presented. **p* <0,05; ***p* <0,01.
